# Prevalence of Carbapenem-Resistant *Klebsiella pneumoniae* Co-Harboring blaKPC-Carrying Plasmid and pLVPK-Like Virulence Plasmid in Bloodstream Infections

**DOI:** 10.3389/fcimb.2020.556654

**Published:** 2021-03-12

**Authors:** Fang-ling Du, Qi-sen Huang, Dan-dan Wei, Yan-fang Mei, Dan Long, Wen-jian Liao, La-gen Wan, Yang Liu, Wei Zhang

**Affiliations:** ^1^ Department of Clinical Microbiology, First Affiliated Hospital of Nanchang University, Nanchang University, Nanchang, China; ^2^ Department of Respiratory, First Affiliated Hospital of Nanchang University, Nanchang University, Nanchang, China

**Keywords:** *Klebsiella pneumonia*, bloodstream infections, pLVPK-like virulence plasmid, KPC-2, carbapenem-resistant

## Abstract

This study aimed to characterize carbapenem-resistant *Klebsiella pneumoniae* (CR-KP) co-harboring *bla*_KPC-2_-carrying plasmid and pLVPK-like virulence plasmid. Between December 2017 and April 2018, 24 CR-KP isolates were recovered from 24 patients with bacteremia. The mortality was 66.7%. Pulsed-field gel electrophoresis and multilocus sequence typing results indicated four clusters, of which cluster A (n = 21, 87.5%) belonged to ST11 and the three remaining isolates (ST412, ST65, ST23) had different pulsotypes (cluster B, C, D). The *bla*_KPC-2_-carrying plasmids all belonged to IncFII_K_ type, and the size ranged from 100 to 390 kb. Nineteen strains (79.2%) had a 219-kb virulence plasmid possessed high similarity to pLVPK from CG43 with serotype K2. Two strains had a 224-kb virulence plasmid resembled plasmid pK2044 from *K. pneumoniae* NTUH-K2044(ST23). Moreover, three strains carried three different hybrid resistance- and virulence-encoding plasmids. Conjugation assays showed that both *bla*_KPC-2_ and *rmpA2* genes could be successfully transferred to *E. coli* J53 in 62.5% of the strains at frequencies of 4.5 × 10^−6^ to 2.4 × 10^−4^, of which three co-transferred *bla*_KPC-2_ along with *rmpA2* in large plasmids. Infection assays in the *Galleria mellonella* model demonstrated the virulence level of these isolates was found to be consistently higher than that of classic *Klebsiella pneumoniae*. In conclusion, CR-KP co-harboring *bla*_KPC-2_-carrying plasmid and pLVPK-like virulence plasmid were characterized by multi-drug resistance, enhanced virulence, and transferability, and should, therefore, be regarded as a real superbug that could pose a serious threat to public health. Hence, heightened efforts are urgently needed to avoid its co-transmission of the virulent plasmid (gene) and resistant plasmid (gene) in clinical isolates.

## Introduction

Carbapenem-resistant *Klebsiella pneumoniae* (CR-KP) has emerged as one of the most challenging pathogens in the latest years (Holt et al., 2015). CR-KP showed resistant to almost all available antibiotics and was related to limited treatment options and high mortality rates. CR-KP has been listed as a “critical priority” by the World Health Organization (WHO). For pathogen survival, the acquisition of virulent traits is necessary (Vila et al., 2011), and some reports suggest that the virulence of carbapenem-resistant *Klebsiella pneumoniae* is enhanced (Ferreira et al., 2018).

The virulence plasmid carrying major virulence genes such as capsular polysaccharides regulator genes (*rmpA* and *rmpA2*) and those encoding siderophores (eg, *iroBCDN*, *iucABCD*, *iutA*) were recognized as essential contributors to the virulence of hypervirulent *Klebsiella pneumoniae* (hvKP), and might serve as potential biomarkers for hvKP. The loss of this pLVPK-derived virulence plasmid significantly decreased virulence. Danxia Gu and colleagues (Gu et al., 2018) reported that CR-KP strains could further evolve to become carbapenem-resistant hvKP (CR-hvKP) through the acquisition of a pLVPK-like virulence plasmid. Meanwhile, CR-hvKP strains may emerge as a result of the acquisition of a carbapenemase-encoding plasmid by K1 or K2 hypervirulent *Klebsiella pneumoniae* (Zhang et al., 2016a). The emergence of carbapenem-resistant hypervirulent *Klebsiella pneumonia*(CR-hvKP) was due to the convergence of virulence and resistance. An increasing number of cases have also been observed worldwide. The high prevalence of carbapenem-resistant *K pneumoniae* (average 9.0% in 2017 and 15.4% in Jiangxi) and hypervirulent *K pneumoniae* (about 30–50%) (Zhang et al., 2016b; Liu and Guo, 2019) in Chinese hospitals may have contributed to the emergence of carbapenem-resistant and hypervirulent microorganism.

In the present study, we characterize clinical characteristics, clonal relationships, virulence and resistance potential of CR-KP co-harboring *bla*_KPC-2_-carrying plasmid and pLVPK-like virulence plasmid in bloodstream infections. The findings of this study provide insight into the current prevalence and features of CR-KP co-harboring *bla*_KPC-2_-carrying plasmid and pLVPK-like virulence plasmid in a Chinese hospital.

## Materials and Methods

### Bacterial Isolates and Antimicrobial Susceptibility Tests

Between December 2017 and April 2018, 24 CR-KP strains, which were identified by the VITEK 2 system (bioMérieux) and confirmed by 16S rRNA gene sequencing, were isolated from blood cultures of 24 patients hospitalized in the First Affiliated Hospital of Nanchang university (Nanchang), Southern China. Antimicrobial susceptibility testing was done for all isolates using Vitek 2 automated systems. Results were interpreted according to the Clinical and Laboratory Standards Institute (document M100-S27). Furthermore, antimicrobial susceptibility of tigecycline was performed by the broth microdilution method and interpreted by the recommendation of the European Committee on Antimicrobial Susceptibility Testing clinical breakpoints (http://www.eucast.org). Patient information was queried from the medical records. This study was approved by the ethical committee of the First Affiliated Hospital of Nanchang University. Informed consent was also obtained from all of the study patients.

### Antimicrobial Resistance Genes

Polymerase chain reaction was used to detect carbapenemase-encoding genes (*bla*_KPC_, *bla*_VIM_, *bla*_NDM_, *bla*_IMP_, and *bla*_OXA-48-like_), β-lactamase genes (*bla*_CTX-M_, *bla*_TEM_, and *bla*_SHV_), plasmid-mediated quinolone resistance determinants (*qnrA, qnrB, qnrS, aac(6′)-Ib-cr*) and 16S rRNA methylase genes(*armA*, *rmtB*) as described previously (Liu et al., 2019). The positive PCR products were purified and sequenced, and the sequences alignments were compared to those in the NCBI database using BLAST.

### Capsular Serotyping and Virulence-Associated Genes Detection

The capsular type of *K. pneumoniae* was determined by PCR and sequencing of *wzi* loci as previously described (Brisse et al., 2013). The sequences of products were compared to the *wzi* sequences deposited in the database of Institute Pasteur to identify the corresponding capsular types using BLAST program (https://bigsdb.pasteur.fr/klebsiella/klebsiella.html). Isolates were screened for the presence of 14 virulence-associated genes,including *rmpA, rmpA2,terW, iutA, silS, mrkD, fimH, ybtS, entB, kpn, aerobactin, kfu, magA*, and *wcaG* (Turton et al., 2018). Primers used for PCR are shown in **Table S2**.

### Plasmid Analysis and Plasmid Transfer

S1 nuclease-pulsed-field gel electrophoresis (S1-PFGE) and southern blotting hybridization were performed to determine the plasmid location of *bla*_KPC-2_-carrying plasmid and virulence plasmid (Xu et al., 2019). Briefly, total DNA was embedded in agarosegel plugs. The plugs were digested with S1 nuclease (TaKaRa) for 30 min at 37°C and then separated by eletrophoresis. Labeling of the probes (**Table S1**) and hybridization were performed with the DIG-High Prime DNA Labeling and Detection Starter Kit II, according to the manufacturer’s instructions (Roche, Basle, Switzerland).

Conjugal transfer experiment was performed using broth-based methods with *Escherichia coli* J53 as the recipient strain. Donor and recipient cells were mixed at 2:1 donor-to-recipient ratio. Transconjugants were selected using 2 or 8 μg/ml potassium tellurite or 2 μg/ml meropenem plus 150 μg/ml sodium azide. Successful conjugation and transformation were confirmed by antimicrobial susceptibility and PCR detection of the *bla*_KPC-2_ gene and pLVPK-derived gene (*rmpA*, *rmpA2*, *terW*, *iutA*, *silS*). S1-PFGE was performed as described previously to confirm acquisition of this plasmid by the recipient strain.

### Galleria Mellonella Infection Model

For virulence testing, the *Galleria mellonella* model was used to investigate toxicity. Ten larvae weighing between 250 and 350 mg (purchased from Tianjin Huiyude Biotech Company, Tianjin, China) were used for the assessment of the virulence level of each isolate. The insects were inoculated by injecting 1 × 10^6^ CFU per 10 µl aliquot into the hemocoel *via* the rear left proleg using methods described previously (Mclaughlin et al., 2014), followed by a recording of survival rate every 12 h for 2 days. All experiments were performed in triplicates. The recent assessment of a range of *K. pneumoniae* isolates suggests the parameters for the *Galleria* model to define hypervirulence, based on a calculation of LD_50_ value (Shi et al., 2018). The hvKP strain NTUH-K2044 and *K. pneumoniae* strain ATCC700603 were used as controls of high and low virulence strains, respectively. Statistical analyses were performed and visualized with GraphPad Prism 7.00.

### Multilocus Sequence Typing (MLST) and Pulsed-Field Gel Electrophoresis (PFGE)

MLST was performed by amplifying and sequencing the seven conserved housekeeping loci including *gapA, infB, mdh, pgi, phoE, rpoB*, and *tonB* (Diancourt et al., 2005), according to protocols on the Pasteur Institute MLST website (http://bigsdb.pasteur.fr/klebsiella/klebsiella.html).

Clonal relatedness was established using XbaI-PFGE (Taraka). DNA fragments were separated with a CHEF DR III apparatus (Bio-Rad; Richmond, CA, USA). The molecular marker was *Salmonella* serotype *Braenderup* strain H9812. The isolates sharing >80% similarity were defined as the same PFGE cluster (Tenover et al., 1995).

## Results

### Patients and Bacterial Isolates

The clinical characteristics of the 24 patients with *K. pneumoniae* bacteremia are shown in **Table 1**. These patients were mainly from the ICU (58.3%, n = 14). The mean age of the patients was 61.9 ± 16.6 years (range, 25–87 years) and 79.2% of these patients were males. The mean time of hospitalization from admission to the identification of CR-KP was 27.5 ± 16.5 days (range, 3–58 days). Hyperglycemia was found among eight cases (33.3%) and seven patients (29.2%) had hypertension. The majority of patients used various invasive procedures and devices, of which the usage rate of mechanical ventilation and tracheal intubation were highest (70.8%). All cases had received a wide variety of antibiotics in combination. The incidence rate of septic shock was 41.7%, and the mortality was 66.7%.

**Table 1:** Clinical characteristics of patients with carbapenem-resistant *K. pneumoniae* bacteremia.

Demographics		Prior antibiotic exposure	
Age (mean ± SD), years	61.9 ± 16.6	Carbapenem	24 (100.0)
Gender, male	19(79.2)	cephalosporin	3 (12.5)
Length of stay (mean ± SD), days	27.5 ± 16.5	β-lactam and β-lactamase inhibitor	11(45.8)
**Underlying disease**		Fluoroquinolone	9 (37.5)
Diabetes mellitus	8(33.3)	Aminoglycoside	8 (33.3)
Hypertension	7(29.2)	Tigecycline	11 (45.8)
**Invasive procedures and devices**		Glycopeptide	14(58.3)
Central venous catheter	13(54.2)	**Clinical outcomes**	
Urinary catheter	14(58.3)	Septic shock	10(41.7)
Endotracheal tube	17(70.8)	30-day Mortality	16(66.7)
Mechanical ventilation	17(70.8)		
Surgical drainage	11(45.8)		
Tracheostomy	5(20.8)		
Surgery	16(66.67)		

### Antimicrobial Susceptibility and Antimicrobial Resistance Genes

The detailed antimicrobial resistance profiles are shown in **Table 2**. The antibiotic susceptibility test showed that all 24 isolates were resistant to ceftriaxone, cefotaxime, aztreonam, ertapenem, imipenem, and meropenem. The percentage of bacteria resistant to gentamicin (16.7%, n = 4), tobramycin (16.7%, n = 4), amikacin (12.5%, n = 3) is low. Resistant to ceftazidime (95.8%, n = 23), cefepime (95.8%, n = 23), piperacillin/tazobactam (91.7%, n = 22), levofloxacin (87.5%, n = 21), ciprofloxacin (87.5%, n = 21), and sulfamethoxazole-trimethoprim (95.8%, n = 23) was high. However, all isolates were sensitive to tigecycline.

**Table 2:** Resistance genes and antibiotic susceptibilities of 24 CR-KP co-harboring *bla*_KPC-2_-carrying plasmid and pLVPK-like virulence plasmid.

Isolates	Resistance profile of *K. pneumoniae*	Carbapenemase	β-lactamase genes		16S rRNA methylase gene	PMQR genes
Kp1	CRO,CAZ,CTX,FEP,TZP,ATM,GEN,TOB,AMK,LVX,CIP,ETP,IMP,MEM,SXT	KPC-2	TEM-1	–		qnrB4
Kp2	CRO,CAZ,CTX,FEP,TZP,ATM,TOB,LVX,CIP,ETP,IMP,MEM,SXT	KPC-2	SHV-12,TEM-1,CTX-M-14	rmtB		qnrS1,acc6-Ib-cr
Kp3	CRO,CAZ,CTX,FEP,TZP,ATM,LVX,CIP,ETP,IMP,MEM	KPC-2	SHV,TEM-1,CTX-M-14	–		acc6-Ib-cr
Kp4	CRO,CAZ,CTX,FEP,TZP,ATM,AMK,LVX,CIP,ETP,IMP,MEM,SXT	KPC-2	SHV-12,TEM-1,CTX-M-14,15	–		qnrS1,acc6-Ib-cr
Kp5	CRO,CAZ,CTX,FEP,TZP,ATM,ETP,IMP,MEM,SXT	KPC-2	SHV-12,TEM-1,CTX-M-14	–		–
Kp6	CRO,CAZ,CTX,FEP,TZP,ATM,LVX,CIP,ETP,IMP,MEM	KPC-2	SHV-11,TEM-1,CTX-M-14	–		qnrS1,acc6-Ib-cr
Kp7	CRO,CAZ,CTX,FEP,TZP,ATM,GEN,TOB,AMK,LVX,CIP,ETP,IMP,MEM,SXT	KPC-2	SHV-12,TEM-1,CTX-M-14	–		qnrS1,acc6-Ib-cr
Kp8	CRO,CAZ,CTX,FEP,TZP,ATM,LVX,CIP,ETP,IMP,MEM,SXT	KPC-2	SHV-12,TEM-1,CTX-M-14	–		qnrS1,acc6-Ib-cr
Kp9	CRO,CAZ,CTX,FEP,TZP,ATM,LVX,CIP,ETP,IMP,MEM,SXT	KPC-2	TEM-1,CTX-M-14	–		qnrS1,acc6-Ib-cr
Kp10	CRO,CAZ,CTX,FEP,TZP,ATM,LVX,CIP,ETP,IMP,MEM,SXT	KPC-2	SHV-11,TEM-1,CTX-M-14	–		qnrS1,acc6-Ib-cr
Kp11	CRO,CAZ,CTX,FEP,TZP,ATM,LVX,CIP,ETP,IMP,MEM,SXT	KPC-2	TEM-1,CTX-M-14	–		qnrS1,acc6-Ib-cr
Kp12	CRO,CAZ,CTX,FEP,TZP,ATM,LVX,CIP,ETP,IMP,MEM,SXT	KPC-2	SHV-12,TEM-1,CTX-M-14	–		qnrB4,qnrS1,acc6-Ib-cr
Kp13	CRO,CAZ,CTX,FEP,TZP,ATM,GEN,LVX,CIP,ETP,IMP,MEM,SXT	KPC-2	SHV-12,TEM-1,CTX-M-14	–		qnrS1,acc6-Ib-cr
Kp14	CRO,CAZ,CTX,FEP,TZP,ATM,LVX,CIP,ETP,IMP,MEM,SXT	KPC-2	SHV-12,TEM-1,CTX-M-14	–		qnrS1,acc6-Ib-cr
Kp15	CRO,CAZ,CTX,FEP,TZP,ATM,LVX,CIP,ETP,IMP,MEM,SXT	KPC-2	TEM-1,CTX-M-14	–		qnrS1
Kp16	CRO,CAZ,CTX,FEP,TZP,ATM,LVX,CIP,ETP,IMP,MEM,SXT	KPC-2	SHV-11,TEM-1,CTX-M-14	–		qnrS1,acc6-Ib-cr
Kp17	CRO,CAZ,CTX,FEP,TZP,ATM,LVX,CIP,ETP,IMP,MEM,SXT	KPC-2	SHV-11,TEM-1,CTX-M-14	–		qnrS1,acc6-Ib-cr
Kp18	CRO,CAZ,CTX,FEP,TZP,ATM,LVX,CIP,ETP,IMP,MEM,SXT	KPC-2	SHV-12,TEM-1,CTX-M-14,-15	–		qnrS1,acc6-Ib-cr
Kp19	CRO,CAZ,CTX,FEP,TZP,ATM,LVX,CIP,ETP,IMP,MEM,SXT	KPC-2	SHV-12,TEM-1,CTX-M-14	–		qnrS1,acc6-Ib-cr
Kp20	CRO,CAZ,CTX,FEP,ATM,LVX,CIP,ETP,IMP,MEM	KPC-2	SHV-11,TEM-1,CTX-M-14	–		qnrS1,acc6-Ib-cr
Kp21	CRO,CAZ,CTX,FEP,TZP,ATM,ETP,IMP,MEM	KPC-2	SHV-12,TEM-1,CTX-M-14	–		qnrS1
Kp22	CRO,CAZ,CTX,FEP,TZP,ATM,GEN,TOB,AMK,LVX,CIP,ETP,IMP,MEM,SXT	KPC-2	SHV-12,TEM-1,CTX-M-14,-15	–		qnrS1
Kp23	CRO,CAZ,CTX,FEP,TZP,ATM,LVX,CIP,ETP,IMP,MEM,TGC,SXT	KPC-2	SHV-12,TEM-1,CTX-M-14	–		qnrS1
Kp24	CRO,CTX,ATM,ETP,IMP,MEM	KPC-2	CTX-M-14	–		–

CRO, ceftriaxone; CAZ, ceftazidime; CTX, cefotaxime; FEP, cefepime; TZP, piperacillin/tazobactam; ATM, Aztreonam; GEN, gentamicin; TOB, Tobramycin; AMK, amikacin; LVX, Levofloxacin; CIP, ciprofloxacin; ETP, Ertapenem; IMP, imipenem; MEM, meropenem; SXT, trimethoprim-sulfamethoxazole.

All the 24 isolates were positive for *bla*_KPC-2_ gene. The β-lactamase genes were detected, including *bla*_TEM-1_ (95.8%, n = 23), *bla*_CTX-M-15_ (95.8%, n = 23), and *bla*_SHV-11_ (79.2%, n = 19). In addition, 20 isolates (83.3%) carried *qnrS1*, 17 isolates carried *aac(6’)-Ib-cr* (70.8%) and 2 isolates (8.3%) carried *qnrB4*. However, only one isolate carried plasmid-mediated 16S rRNA methylase gene *rmtB*. All isolates were negative for *bla*_VIM_, *bla*_NDM_, *bla*_IMP_, *bla*_OXA-48_, *armA*, and *qnrS*.

### Plasmid Profiles

**Figure 1:**
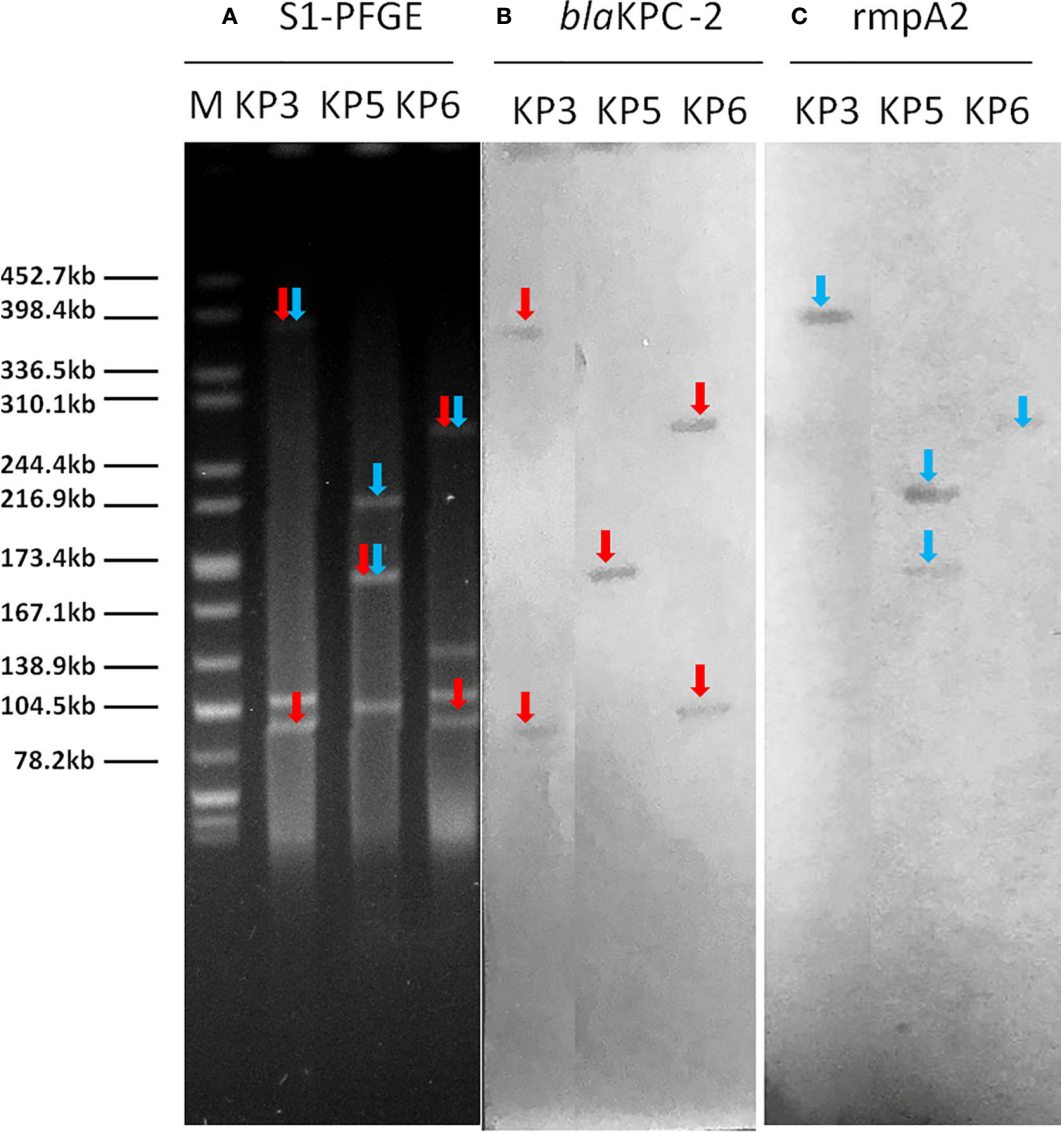
The S1-PFGE and Southern hybridization analysis of 3 strains hybrid resistance- and virulence-encoding plasmids. Notes: **(A)** S1 nuclease digestion of genomic DNA of *K. pneumoniae* strains was followed by PFGE. Plasmid bands are shown as linearized fragment on the gel. **(B)** Southern blot hybridization of *bla*_KPC-2_ gene. Mark it with a red arrow. **(C)** Southern blot hybridization of the marker gene (rmpA2) of the virulence plasmid. Mark it with a blue arrow. Lane M, reference standard strain *Salmonella* serotype Braenderup H9812 restricted with Xbal. M, marker; S1-PFGE, S1 nuclease pulsed-field gel electrophoresis.

Plasmid location of *bla*_KPC_-carrying plasmid and pLVPK-like virulence plasmid was determined by S1-PFGE and Southern blot analysis. The results demonstrated that the plasmid size carrying *bla*_KPC-2_ ranged from 100 to 390 kb (**Figures 1**, **S1**). Furthermore, two isolates had two different plasmids harboring *bla*_KPC-2_ gene. Nineteen strains (79.2%) had a 219-kb virulence plasmid possessed high similarity to previously reported pLVPK from *Klebsiella pneumoniae* CG43 with serotype K2. Two strains had a 224-kb virulence plasmid resembled plasmid pK2044 from *K. pneumoniae* NTUH-K2044 belonged to sequence type 23. Moreover, there were three isolates (KP3, KP5, KP6) carrying a hybrid resistance- and virulence-encoding plasmid, which harbored both the carbapenemase gene *bla*_KPC-2_ and the virulence gene *rmpA2*.

Conjugation assays showed that both *bla*_KPC-2_ and *rmpA2* genes could be successfully transferred to *E. coli* J53 in 62.5% (15/24) of the strains at frequencies of 4.5 × 10^−6^ to 2.4 × 10^−4^ (transconjugant/recipient), of which three co-transferred *bla*_KPC-2_ along with *rmpA2* in large plasmids. KP3 isolate transferred a hybrid resistance- and virulence-encoding plasmid of 390 kb to *E. coli* J53 at a frequency of 3.5 × 10^−5^ (transconjugant/recipient) by mating. In addition, KP10 and KP24 isolates co-transferred the *bla*_KPC-2_-carrying plasmid and pLVPK-like virulence plasmid to *E. coli* J53 at a frequency of 7.4 × 10^−6^ (transconjugant/recipient) by mating (**Table S3**).

### Virulence-Associated Features

**Figure 2:**
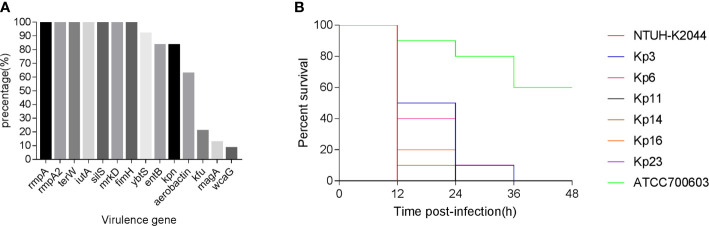
**(A)** The distributions of virulence-associated genes among CR-KP co-harboring blaKPC-2-carrying plasmid and pLVPK-like virulence plasmid. **(B)** Virulence potential of representative CR-KP co-harboring *bla*_KPC-2_-carrying plasmid and pLVPK-like virulence plasmid in a *Galleria mellonella* infection model. CR-KP, carbapenem-resistant *Klebsiella pneumoniae*.

The prevalence and distribution of virulence factors are shown in **Figure 2A**. The virulence-related genes detected in 24 isolates included *fimH-1* (100%), *mrkD* (100%), *ybtS* (91.7%), *entB* (83.3%), *kpn* (83.3%), *aerobactin* (62.5%), *kfu* (20.8%), *magA* (12.5%), and *wcaG* (8.3%) (**Figure S2**). Moreover, all the five pLVPK-derived locus, *rmpA, rmpA2, terW, iutA, silS*, were detected in all 24 isolates.

The *G. mellonella* larvae infection model was used to assess the potential virulence of these isolates (**Figure 2B**). After 48 h of infection, the mortality of the larvae infected with CR-KP isolates co-carrying virulence plasmid and KPC-2 plasmid were consistently higher than that infected with cKP (*P* < 0.05) (**Table S2**). Among the 24 strains, the virulence level of 15 isolates is similar to hvKP previously reported (P>0.05), but nine isolates are less virulent (P < 0.05) (**Table S2**).

### Clonal Relationship

**Figure 3:**
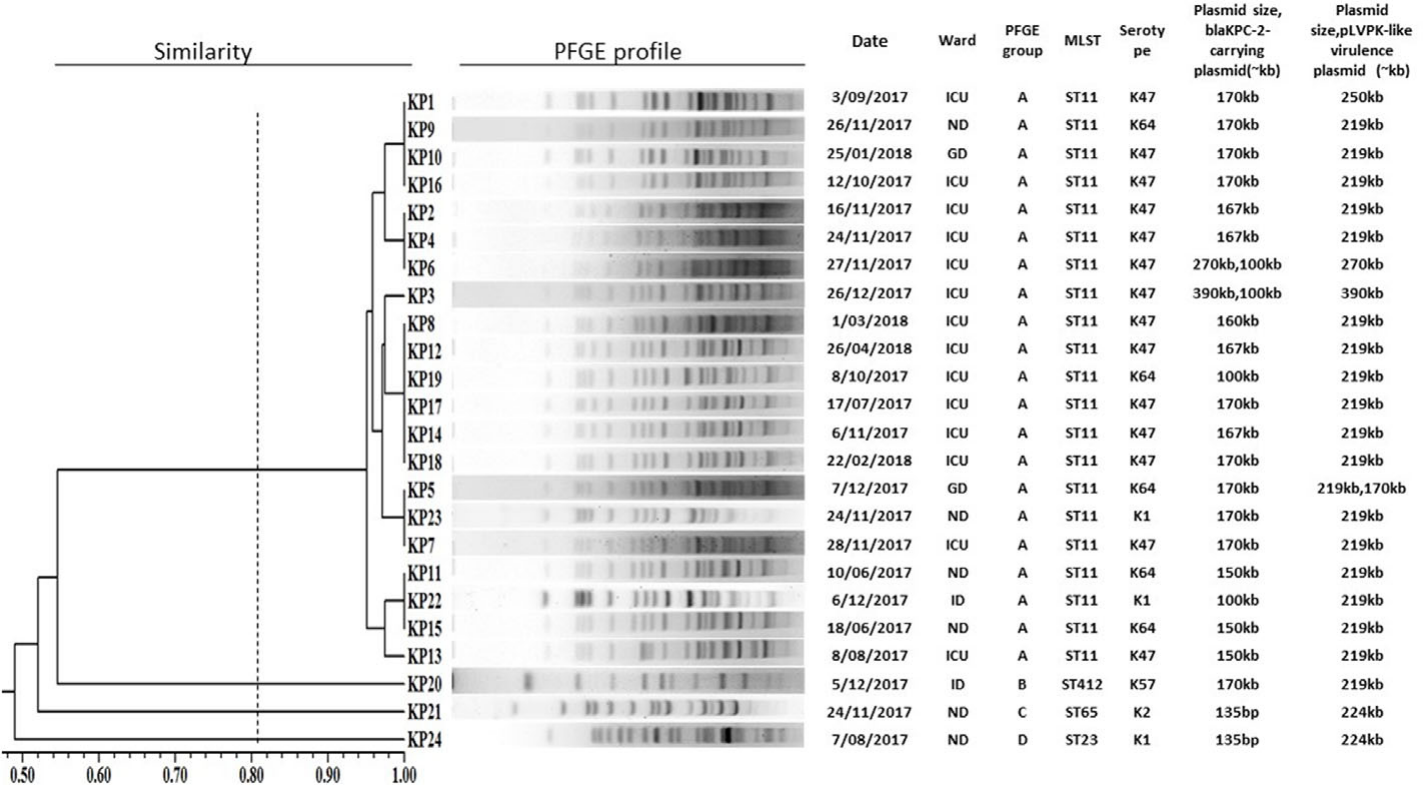
PFGE dendrogram of 24 carbapenem-resistant *Klebsiella pneumoniae* co-harboring *bla*_KPC-2_-carrying plasmid and pLVPK-like virulence plasmid. ICU, intensive care unit; ND, neurosurgery department; GD, gastroenterology department; ID, infectious department.

Among the 24 isolates, four STs were identified, including ST11 (14 wzi47-K47 isolates, five wzi64-K64 isolates, and two wzi125-K1 isolates), ST23 (1 wzi1-K1 isolate), ST65 (1 wzi2-K2 isolate), ST412 (1 wzi206-K57 isolate). PFGE (**Figure 1**) identified one major pulsotype (cluster A), encompassing 21 of the 24 isolates, all belonging to ST11 (**Figure 3**). The three remaining isolates (Kp20, Kp21, and Kp24) had different pulsotypes.

## Discussion

In our study, we reported the prevalence of carbapenem-resistant *K. pneumoniae* co-harboring *bla*_KPC-2_-carrying plasmid and pLVPK-like virulence plasmid in patients with bacteremia. *Klebsiella pneumoniae* is the second most common pathogen in *Enterobacteriaceae* bloodstream infections (Meatherall et al., 2009). In this study, the overall 30-day mortality rate was 66.7%, which was higher than in those with KPC-producing *K. pneumoniae* bloodstream infections (44.2%) (Xu et al., 2018). Ten patients (41.7%) developed septic shock, which was the recognized reason for increased mortality (Falcone et al., 2016). In addition, 33.3% of the patients had hyperglycemia, which was considered to be a significant risk factor for hypervirulent *Klebsiella pneumoniae* infection (Zhang et al., 2016b). There are many possible contributing factors to the emergence, rise, and spread of antibiotic resistance, including ICU admission, antibiotics exposure, using invasive devices and procedures (Li et al., 2019). These risk factors may have contributed to the high rates of antibiotic resistance found in our study.

Although all the 24 CR-KP co-harboring *bla*_KPC-2_-carrying plasmid and pLVPK-like virulence plasmid were multi-drug-resistant, amikacin, gentamicin, and tigecycline still had efficient antimicrobial activity *in vitro* against these isolates, indicating that they could be valuable treatment choices. The production of *Klebsiella pneumoniae* carbapenemase (KPC) is the most prevalent mechanism of resistance to carbapenems (Munoz-Price et al., 2013). In China, the first detection of the plasmid-mediated class A carbapenemase KPC-2 gene was located on an approximately 60-kb plasmid in 2007 (Wei et al., 2007). In this study, the *bla*_KPC-2_ carrying plasmids all belonged to IncFII_K_ type, and the size ranged from 100-kb to 390-kb.

Virulence plasmids were associated with hypervirulent serotypes of *Klebsiella pneumoniae* and predisposed patients to abscess formation (Tang et al., 2010). In the present study, nineteen strains (79.2%) carry a 219-kb virulence plasmid similar to pLVPK plasmid from serotype K2, *K. pneumoniae* CG43 (Chen et al., 2004). Two strains (8.3%) carry a 224-kb virulence plasmid similar to the pK2044 plasmid from serotype K1, sequence type (ST) 23 strain NTUH-K2044 (Wu et al., 2009). The pLVPK-like virulence plasmids in *K. pneumoniae* are very large and would, therefore, be regarded as non-conjugative. This would explain their strong association with particular hypervirulent serotypes (Struve et al., 2015). Nevertheless, it is obvious that virulence plasmids have been reported in several serotypes of *Klebsiella*, indicating that conjugation is occurring, albeit at a low frequency. In this study, three strains CR-KP co-harboring *bla*_KPC-2_-carrying plasmid and pLVPK-like virulence plasmid can transfer virulence plasmids to *E. coli* J53. The conjugative transfer of this virulence plasmid increased the virulence level of such strain.

Carbapenem-resistant *K. pneumoniae* rarely carry virulence plasmids and hypervirulent *K. pneumoniae* generally do not carry antibiotic resistance genes. Nevertheless, in the current study, 24 strains *Klebsiella pneumoniae* co- harbored *bla*_KPC-2_-carrying plasmid and pLVPK-like virulence plasmid. Most recently, Dong et al. (2018) reported that a *bla*_KPC-2_-encoding element can be integrated into a virulence plasmid, which then possesses the ability to mediate expression of both hypervirulence and hyper-resistance phenotype in K1 hypervirulent *Klebsiella pneumoniae*. Similarly, we found three strains ST11 *K. pneumoniae* carrying a *bla*_KPC-2_-harboring virulence plasmid, which were approximately 390, 270, and 170 kb, respectively. The convergence of virulence and MDR in a single plasmid vector enables simultaneous transfer and potentially rapid emergence of hypervirulence-MDR *K. pneumoniae* clones.

The presence of *mrkD* and *fimH* has previously been related to KPC-positive *K. pneumoniae* (De Cassia Andrade Melo et al., 2014). However, previous studies (Yeh et al., 2007) reported that *magA* was characteristic of the K1 capsular operon, which was associated with the hypermucoviscosity phenotype of *K. pneumoniae*. Siderophore-associated genes, such as *entB*, *ybtS*, and *iutA*, were critical for bacterial growth, replication, and virulence (Holden and Bachman, 2015). *entB* was only characterized for virulence when it occurs in association with *iutA* or *ybtS* (Daehre et al., 2018). By analyzing virulence genes, all *K. pneumoniae* isolates carried both *mrkD a*nd *fimH* genes in our study. Moreover, the *entB, iutA* or *ybtS* genes were present from three-quarters of all isolates, all of which serve as high mark of virulence.

Capsule, lipopolysaccharide (LPS), fimbriae (types 1 and 3), siderophores, and pLVPK-like virulence plasmid are virulence factors that contribute to the pathogenicity of *K. pneumoniae*. Nevertheless, Shu et al. (2019) reported OXA-232-producing ST15 carbapenem-resistant *K. pneumoniae* were not hypervirulent despite harboring a virulence plasmid. In the current study, the virulence level of CR-KP co-harboring *bla*_KPC-2_-carrying plasmid and pLVPK-like virulence plasmid was found to be consistently higher than that of cKP. But we also found nine strains CR-KP co-harboring *bla*_KPC-2_-carrying plasmid and pLVPK-like virulence plasmid were less virulent than hvKP. Further studies are required to establish the relationship between the hypervirulence phenotype and the carriage of the virulence plasmid in *K. pneumoniae*.

In our study, 87.5% of CR-KP co-harboring *bla*_KPC-2_-carrying plasmid and pLVPK-like virulence plasmid belonged to ST11, in accordance with the report by Qi et al. (2011), which described that ST11 was the dominant clone of KPC-2-producing *K. pneumoniae* in China. Nineteen out of twenty-one ST11 isolates were wzi47-K47 or wzi64-K64 by the capsular serotyping. Two ST11 isolates belonged to wzi125-K1, which was rarely reported in a previous study (Wei et al., 2016). One wzi1-K1 strain belonged to ST23, was strongly correlated with liver abscess (Shon et al., 2013); one wzi2-K2 strain belonged to ST65, which is in accordance with the previous study that ST65 was the most common ST associated with K2 serotype in *K. pneumoniae* (Liao et al., 2014); one wzi206-K57 belonged to ST412, which was hypermucoviscous.

In conclusion, all isolates were characterized by multi-drug resistance, enhanced virulence, and transferability, and should, therefore, be regarded as a real superbug that could pose a serious threat to public health. Moreover, three strains carried 3 different hybrid resistance- and virulence-encoding plasmids. We should strengthen the ability of anti-infective prophylaxis and management to avoid its co-transmission of the virulent plasmid (gene) and resistant plasmid (gene) in clinical isolates.

## Data Availability Statement

The raw data supporting the conclusions of this article will be made available by the authors, without undue reservation.

## Ethics Statement

This study was approved by the ethical committee of the First Affiliated Hospital of Nanchang University. Informed consent was also obtained from all of the study patients.

## Author Contributions

F-lD, Q-sH, D-dW, and YL conceived and designed the experiments. F-lD, Q-sH, D-dW, DL, and W-jL designed and performed the experiments. F-lD, L-gW, and WZ analyzed the data. F-lD and YL wrote the manuscript. YL contributed to review on data analysis and the interpretation of the data. All authors contributed to the article and approved the submitted version.

## Funding

This study was supported by the National Natural Science Foundation of China (81560323), Education Department of Jiangxi Province, China (180016), Jiangxi science and Technology Department in China (20202ZDB01016, 20202ACBL206025, 20181BAB205065 and 20202ACBL206023) and Health and Family Planning Commission of Jiangxi Province (20188006 and 2018A330).

## Conflict of Interest

The authors declare that the research was conducted in the absence of any commercial or financial relationships that could be construed as a potential conflict of interest.

## References

[B1] Brisse S. Passet V. Haugaard A. B. Babosan A. Kassis-Chikhani N. Struve C.. (2013). wzi Gene sequencing, a rapid method for determination of capsular type for Klebsiella strains. J. Clin. Microbiol. 51, 4073–4078. 10.1128/JCM.01924-13 24088853 PMC3838100

[B2] Chen Y. T. Chang H. Y. Lai Y. C. Pan C. C. Tsai S. F. Peng H. L. (2004). Sequencing and analysis of the large virulence plasmid pLVPK of Klebsiella pneumoniae CG43. Gene 337, 189–198. 10.1016/j.gene.2004.05.008 15276215

[B3] Daehre K. Projahn M. Friese A. Semmler T. Guenther S. Roesler U. H. (2018). ESBL-Producing Klebsiella pneumoniae in the Broiler Production Chain and the First Description of ST3128. Front. Microbiol. 9, 2302–2307. 10.3389/fmicb.2018.02302 30337912 PMC6178893

[B4] De Cassia Andrade Melo R. De Barros E. M. Loureiro N. G. De Melo H. R. Maciel M. A. Souza Lopes A. C. (2014). Presence of fimH, mrkD, and irp2 virulence genes in KPC-2-producing Klebsiella pneumoniae isolates in Recife-PE, Brazil. Curr. Microbiol. 69, 824–831. 10.1007/s00284-014-0662-0 25085544

[B5] Diancourt L. Passet V. Verhoef J. Grimont P. A. Brisse S. (2005). Multilocus sequence typing of Klebsiella pneumoniae nosocomial isolates. J. Clin. Microbiol. 43, 4178–4182. 10.1128/JCM.43.8.4178-4182.2005 16081970 PMC1233940

[B6] Dong N. Lin D. Zhang R. Chan E. W. Chen S. (2018). Carriage of blaKPC-2 by a virulence plasmid in hypervirulent Klebsiella pneumoniae. J. Antimicrob. Chemother. 73, 3317–3321. 10.1093/jac/dky358 30239821

[B7] Falcone M. Russo A. Iacovelli A. Restuccia G. Ceccarelli G. Giordano A.. (2016). Predictors of outcome in ICU patients with septic shock caused by Klebsiella pneumoniae carbapenemase-producing K. pneumoniae. Clin. Microbiol. Infect. 22, 444–450. 10.1016/j.cmi.2016.01.016 26850826

[B8] Ferreira R. L. Da Silva B. C. M. Rezende G. S. Nakamura-Silva R. Pitondo-Silva A. Campanini E. B.. (2018). High Prevalence of Multidrug-Resistant Klebsiella pneumoniae Harboring Several Virulence and beta-Lactamase Encoding Genes in a Brazilian Intensive Care Unit. Front. Microbiol. 9, 3198. 10.3389/fmicb.2018.03198 30723463 PMC6349766

[B9] Gu D. Dong N. Zheng Z. Lin D. Huang M. Wang L.. (2018). A fatal outbreak of ST11 carbapenem-resistant hypervirulent Klebsiella pneumoniae in a Chinese hospital: a molecular epidemiological study. Lancet Infect. Dis. 18, 37–46. 10.1016/S1473-3099(17)30489-9 28864030

[B10] Holden V. I. Bachman M. A. (2015). Diverging roles of bacterial siderophores during infection. Metallomics 7, 986–995. 10.1039/C4MT00333K 25745886

[B11] Holt K. E. Wertheim H. Zadoks R. N. Baker S. Whitehouse C. A. Dance D.. (2015). Genomic analysis of diversity, population structure, virulence, and antimicrobial resistance in Klebsiella pneumoniae, an urgent threat to public health. Proc. Natl. Acad. Sci. U. S. A. 112, E3574–E3581. 10.1073/pnas.1501049112 26100894 PMC4500264

[B12] Li J. Li Y. Song N. Chen Y. (2019). Risk factors for carbapenem-resistant Klebsiella pneumoniae infection: a meta-analysis. J. Glob. Antimicrob. Resist. 21, 306–313. 10.1016/j.jgar.2019.09.006 31525540

[B13] Liao C. H. Huang Y. T. Chang C. Y. Hsu H. S. Hsueh P. R. (2014). Capsular serotypes and multilocus sequence types of bacteremic Klebsiella pneumoniae isolates associated with different types of infections. Eur. J. Clin. Microbiol. Infect. Dis. 33, 365–369. 10.1007/s10096-013-1964-z 24013597

[B14] Liu C. Guo J. (2019). Hypervirulent Klebsiella pneumoniae (hypermucoviscous and aerobactin positive) infection over 6 years in the elderly in China: antimicrobial resistance patterns, molecular epidemiology and risk factor. Ann. Clin. Microbiol. Antimicrob. 18, 4. 10.1186/s12941-018-0302-9 30665418 PMC6341648

[B15] Liu Y. Du F. L. Xiang T. X. Wan L. G. Wei D. D. Cao X. W.. (2019). High Prevalence of Plasmid-Mediated Quinolone Resistance Determinants Among Serotype K1 Hypervirulent Klebsiella pneumoniae Isolates in China. Microb. Drug Resist. 10.1089/mdr.2018.0173 30615560

[B16] Mclaughlin M. M. Advincula M. R. Malczynski M. Barajas G. Qi C. Scheetz M. H. (2014). Quantifying the clinical virulence of Klebsiella pneumoniae producing carbapenemase Klebsiella pneumoniae with a Galleria mellonella model and a pilot study to translate to patient outcomes. BMC Infect. Dis. 14, 31. 10.1186/1471-2334-14-31 24428847 PMC3897888

[B17] Meatherall B. L. Gregson D. Ross T. Pitout J. D. Laupland K. B. (2009). Incidence, risk factors, and outcomes of Klebsiella pneumoniae bacteremia. Am. J. Med. 122, 866–873. 10.1016/j.amjmed.2009.03.034 19699383

[B18] Munoz-Price L. S. Poirel L. Bonomo R. A. Schwaber M. J. Daikos G. L. Cormican M.. (2013). Clinical epidemiology of the global expansion of Klebsiella pneumoniae carbapenemases. Lancet Infect. Dis. 13, 785–796. 10.1016/S1473-3099(13)70190-7 23969216 PMC4673667

[B19] Qi Y. Wei Z. Ji S. Du X. Shen P. Yu Y. (2011). ST11, the dominant clone of KPC-producing Klebsiella pneumoniae in China. J. Antimicrob. Chemother. 66, 307–312. 10.1093/jac/dkq431 21131324

[B20] Shi Q. Lan P. Huang D. Hua X. Jiang Y. Zhou J.. (2018). Diversity of virulence level phenotype of hypervirulent Klebsiella pneumoniae from different sequence type lineage. BMC Microbiol. 18, 94. 10.1186/s12866-018-1236-2 30157774 PMC6116568

[B21] Shon A. S. Bajwa R. P. Russo T. A. (2013). Hypervirulent (hypermucoviscous) Klebsiella pneumoniae: a new and dangerous breed. Virulence 4, 107–118. 10.4161/viru.22718 23302790 PMC3654609

[B22] Shu L. Dong N. Lu J. Zheng Z. Hu J. Zeng W.. (2019). Emergence of OXA-232 Carbapenemase-Producing Klebsiella pneumoniae That Carries a pLVPK-Like Virulence Plasmid among Elderly Patients in China. Antimicrob. Agents Chemother. 63 (3), e02246–18. 10.1128/AAC.02246-18 PMC6395905 30559135

[B23] Struve C. Roe C. C. Stegger M. Stahlhut S. G. Hansen D. S. Engelthaler D. M.. (2015). Mapping the Evolution of Hypervirulent Klebsiella pneumoniae. MBio 6, e00630. 10.1128/mBio.00630-15 26199326 PMC4513082

[B24] Tang H. L. Chiang M. K. Liou W. J. Chen Y. T. Peng H. L. Chiou C. S.. (2010). Correlation between Klebsiella pneumoniae carrying pLVPK-derived loci and abscess formation. Eur. J. Clin. Microbiol. Infect. Dis. 29, 689–698. 10.1007/s10096-010-0915-1 20383552

[B25] Tenover F. C. Arbeit R. D. Goering R. V. Mickelsen P. A. Murray B. E. Persing D. H.. (1995). Interpreting chromosomal DNA restriction patterns produced by pulsed-field gel electrophoresis: criteria for bacterial strain typing. J. Clin. Microbiol. 33, 2233–2239. 10.1128/JCM.33.9.2233-2239.1995 7494007 PMC228385

[B26] Turton J. F. Payne Z. Coward A. Hopkins K. L. Turton J. A. Doumith M.. (2018). Virulence genes in isolates of Klebsiella pneumoniae from the UK during 2016, including among carbapenemase gene-positive hypervirulent K1-ST23 and ‘non-hypervirulent’ types ST147, ST15 and ST383. J. Med. Microbiol. 67, 118–128. 10.1099/jmm.0.000653 29205138

[B27] Vila A. Cassata A. Pagella H. Amadio C. Yeh K. M. Chang F. Y.. (2011). Appearance of Klebsiella pneumoniae liver abscess syndrome in Argentina: case report and review of molecular mechanisms of pathogenesis. Open Microbiol. J. 5, 107–113. 10.2174/1874285801105010107 22145012 PMC3229087

[B28] Wei Z. Q. Du X. X. Yu Y. S. Shen P. Chen Y. G. Li L. J. (2007). Plasmid-mediated KPC-2 in a Klebsiella pneumoniae isolate from China. Antimicrob. Agents Chemother. 51, 763–765. 10.1128/AAC.01053-06 17145797 PMC1797727

[B29] Wei D. D. Wan L. G. Deng Q. Liu Y. (2016). Emergence of KPC-producing Klebsiella pneumoniae hypervirulent clone of capsular serotype K1 that belongs to sequence type 11 in Mainland China. Diagn. Microbiol. Infect. Dis. 85, 192–194. 10.1016/j.diagmicrobio.2015.03.012 27049969

[B30] Wu K. M. Li L. H. Yan J. J. Tsao N. Liao T. L. Tsai H. C.. (2009). Genome sequencing and comparative analysis of Klebsiella pneumoniae NTUH-K2044, a strain causing liver abscess and meningitis. J. Bacteriol. 191, 4492–4501. 10.1128/JB.00315-09 19447910 PMC2704730

[B31] Xu M. Fu Y. Kong H. Chen X. Chen Y. Li L.. (2018). Bloodstream infections caused by Klebsiella pneumoniae: prevalence of blaKPC, virulence factors and their impacts on clinical outcome. BMC Infect. Dis. 18, 358. 10.1186/s12879-018-3263-x 30064360 PMC6069789

[B32] Xu M. Fu Y. Fang Y. Xu H. Kong H. Liu Y.. (2019). High prevalence of KPC-2-producing hypervirulent Klebsiella pneumoniae causing meningitis in Eastern China. Infect. Drug Resist. 12, 641–653. 10.2147/IDR.S191892 30936727 PMC6430001

[B33] Yeh K. M. Kurup A. Siu L. K. Koh Y. L. Fung C. P. Lin J. C.. (2007). Capsular serotype K1 or K2, rather than magA and rmpA, is a major virulence determinant for Klebsiella pneumoniae liver abscess in Singapore and Taiwan. J. Clin. Microbiol. 45, 466–471. 10.1128/JCM.01150-06 17151209 PMC1829066

[B34] Zhang R. Lin D. Chan E. W. Gu D. Chen G. X. Chen S. (2016a). Emergence of Carbapenem-Resistant Serotype K1 Hypervirulent Klebsiella pneumoniae Strains in China. Antimicrob. Agents Chemother. 60, 709–711. 10.1128/AAC.02173-15 26574010 PMC4704206

[B35] Zhang Y. Zhao C. Wang Q. Wang X. Chen H. Li H.. (2016b). High Prevalence of Hypervirulent Klebsiella pneumoniae Infection in China: Geographic Distribution, Clinical Characteristics, and Antimicrobial Resistance. Antimicrob. Agents Chemother. 60, 6115–6120. 10.1128/AAC.01127-16 27480857 PMC5038323

