# Supplementary material for: Prevalence of Carbapenem-Resistant Klebsiella pneumoniae Co-Harboring blaKPC-Carrying Plasmid and pLVPK-Like Virulence Plasmid in Bloodstream Infections
Source: Front Cell Infect Microbiol. 2021 Mar 12;10:556654. doi: 10.3389/fcimb.2020.556654 (PMC7996060; doi:10.3389/fcimb.2020.556654)
Supplement: Supplementary file 1 [file DataSheet_1.docx]

Supplementary data

Table S1. Primers used to detect the virulence-associated genes in this study. F indicates the forward primer and R indicates the reverse primer.

Table S2. Primers for southern blot in this study. F indicates the forward primer and R indicates the reverse primer.

Table S3. Virulence potential of carbapenem-resistant Klebsiella pneumoniae strains in a Galleria mellonella infection model

Table S4. Minimum inhibitory concentrations, associated markers and plasmid size of 3 strains carbapenem-resistant *Klebsiella pneumoniae* co-transferring *bla*KPC-2-carrying plasmid and pLVPK-like virulence plasmid, its transconjugants, and *Escherichia coli* J53

Figure S1. The S1-PFGE of 24 carbapenem-resistant Klebsiella pneumoniae strains

Table S1. Primers used to detect the virulence-associated genes in this study

| Primer name | Sequence (5’-3’) | Product size |
| --- | --- | --- |
| rmpA-F | ACGACTTTCAAGAGAAATGA | 434bp |
| rmpA-R | CATAGATGTCATAATCACAC |  |
| rmpA2-F | CTTTATGTGCAATAAGGATGTT | 452bp |
| rmpA2-R | CCTCCTGGAGAGTAAGCATT |  |
| terW-F | ATGCAATTAAACACCAGACAG | 239bp |
| terW-R | CTCATTCTCTTGAGTGTTTTC |  |
| iutA-F | ACCTGGGTTATCGAAAACGC | 1115bp |
| iutA-R | GATGTCATAGCCTGATTGC |  |
| silS-F | CATAGCAAACCTTCCAGGC | 803bp |
| silS-R | ATCGGCAGAGAAATTGGC |  |
| mrkD-F | AAGCTATCGCTGTACTTCCGGCA | 340bp |
| mrkD-R | GGCGTTGGCGCTCAGATAGG |  |
| fimH-F | TGCTGCTGGGCTGGTCGATG | 909bp |
| fimH-R | GGGAGGGTGACGGTGACATC |  |
| ybtS-F | GACGGAAACAGCACGGTAAA | 782bp |
| ybtS-R | GAGCATAATAAGGCGAAAGA |  |
| entB-F | GTCAACTGGGCCTTTGAGCCGTC | 400bp |
| entB-R | TATGGGCGTAAACGCCGGTGAT |  |
| kpn-F | GTATGACTCGGGGAAGATTA | 628bp |
| kpn-R | CAGAAGCAGCCACCACG |  |
| aerobactin-F | GCATAGGCGGATACGAACAT | 556bp |
| aerobactin-R | CACAGGGCAATTGCTTACCT |  |
| kfu-F | ATAGTAGGCGAGCACCGAGA | 530bp |
| kfu-R | AGAACCTTCCTCGCTGAACA |  |
| magA-F | GGTGCTCTTTACATCATTGC | 1283bp |
| magA-R | ATAGTAGGCGAGCACCGAGA |  |
| wcaG-F | GGTTGGKTCAGCAATCGTA | 169bp |
| wcaG-R | ACTATTCCGCCAACTTTTGC |  |

Table S2. Primers for southern blot in this study

| Primer name | Sequence (5’-3’) | Product size |
| --- | --- | --- |
| KPC-2-F | TGTAAGTTACCGCGCTGAGG | 582bp |
| KPC-2-R | CCAGACGACGGCATAGTCAT |  |
| rmpA2-F | CTTTATGTGCAATAAGGATGTT | 452bp |
| rmpA2-R | CCTCCTGGAGAGTAAGCATT |  |

Table S3. Virulence potential of carbapenem-resistant Klebsiella pneumoniae strains in a Galleria mellonella infection model

| Isolates | 0h | 12h | 24h | 36h | 48h | P-value(vs NTUH-K2044) | P-value(vs cKP) |  |
| --- | --- | --- | --- | --- | --- | --- | --- | --- |
| KP1 | 10 | 2 | 0 | 0 | 0 | P>0.05 | P<0.05 | high |
| KP2 | 10 | 4 | 1 | 0 | 0 | P<0.05 | P<0.05 | middle |
| KP3 | 10 | 5 | 1 | 0 | 0 | P<0.05 | P<0.05 | middle |
| KP4 | 10 | 4 | 1 | 0 | 0 | P<0.05 | P<0.05 | middle |
| KP5 | 10 | 2 | 0 | 0 | 0 | P>0.05 | P<0.05 | high |
| KP6 | 10 | 4 | 1 | 0 | 0 | P<0.05 | P<0.05 | middle |
| KP7 | 10 | 2 | 0 | 0 | 0 | P>0.05 | P<0.05 | high |
| KP8 | 10 | 1 | 0 | 0 | 0 | P>0.05 | P<0.05 | high |
| KP9 | 10 | 2 | 0 | 0 | 0 | P>0.05 | P<0.05 | high |
| KP10 | 10 | 2 | 0 | 0 | 0 | P>0.05 | P<0.05 | high |
| KP11 | 10 | 4 | 0 | 0 | 0 | P<0.05 | P<0.05 | middle |
| KP12 | 10 | 1 | 0 | 0 | 0 | P>0.05 | P<0.05 | high |
| KP13 | 10 | 4 | 0 | 0 | 0 | P<0.05 | P<0.05 | middle |
| KP14 | 10 | 1 | 0 | 0 | 0 | P>0.05 | P<0.05 | high |
| KP15 | 10 | 4 | 0 | 0 | 0 | P<0.05 | P<0.05 | middle |
| KP16 | 10 | 2 | 0 | 0 | 0 | P>0.05 | P<0.05 | high |
| KP17 | 10 | 1 | 0 | 0 | 0 | P>0.05 | P<0.05 | high |
| KP18 | 10 | 1 | 0 | 0 | 0 | P>0.05 | P<0.05 | high |
| KP19 | 10 | 1 | 0 | 0 | 0 | P>0.05 | P<0.05 | high |
| KP20 | 10 | 5 | 1 | 0 | 0 | P<0.05 | P<0.05 | middle |
| KP21 | 10 | 1 | 0 | 0 | 0 | P>0.05 | P<0.05 | high |
| KP22 | 10 | 4 | 0 | 0 | 0 | P<0.05 | P<0.05 | middle |
| KP23 | 10 | 2 | 0 | 0 | 0 | P>0.05 | P<0.05 | high |
| KP24 | 10 | 1 | 0 | 0 | 0 | P>0.05 | P<0.05 | high |
| NTUH-K2044 | 10 | 0 | 0 | 0 | 0 |  |  |  |
| ATCC  700603 | 10 | 9 | 8 | 6 | 6 |  |  |  |

Table S4. Minimum inhibitory concentrations, associated markers and plasmid size of 3 strains carbapenem-resistant Klebsiella pneumoniae co-transferring *bla*KPC-2-carrying plasmid and pLVPK-like virulence plasmid, its transconjugants, and *Escherichia coli* J53

| Isolate name and transconjugate | Meropenem MIC (ug/mL) | Organism name | Associated markers | | | | | | Plasmid size (kb) |
| --- | --- | --- | --- | --- | --- | --- | --- | --- | --- |
|  |  |  | *KPC-2* | *rmpA2* | *rmpA* | *iutA* | *terW* | *silS* |  |
| KP3 | 128 | Klebsiella pneumoniae | + | + | + | + | + | + | 390kb,105kb,100kb |
| KP3-T | 64 | KP3-T Escherichia coli J53 | + | + | + | + | + | + | 390kb |
| KP10 | 64 | Klebsiella pneumoniae | + | + | + | + | + | + | 219kb,170kb,105kb |
| KP10-T | 64 | KP10-T Escherichia coli J53 | + | + | + | + | + | + | 219kb,170kb |
| KP24 | 16 | Klebsiella pneumoniae | + | + | + | + | + | + | 224kp,135kb |
| KP24-T | 16 | KP24-T Escherichia coli J53 | + | + | + | + | + | + | 224kp,135kb |


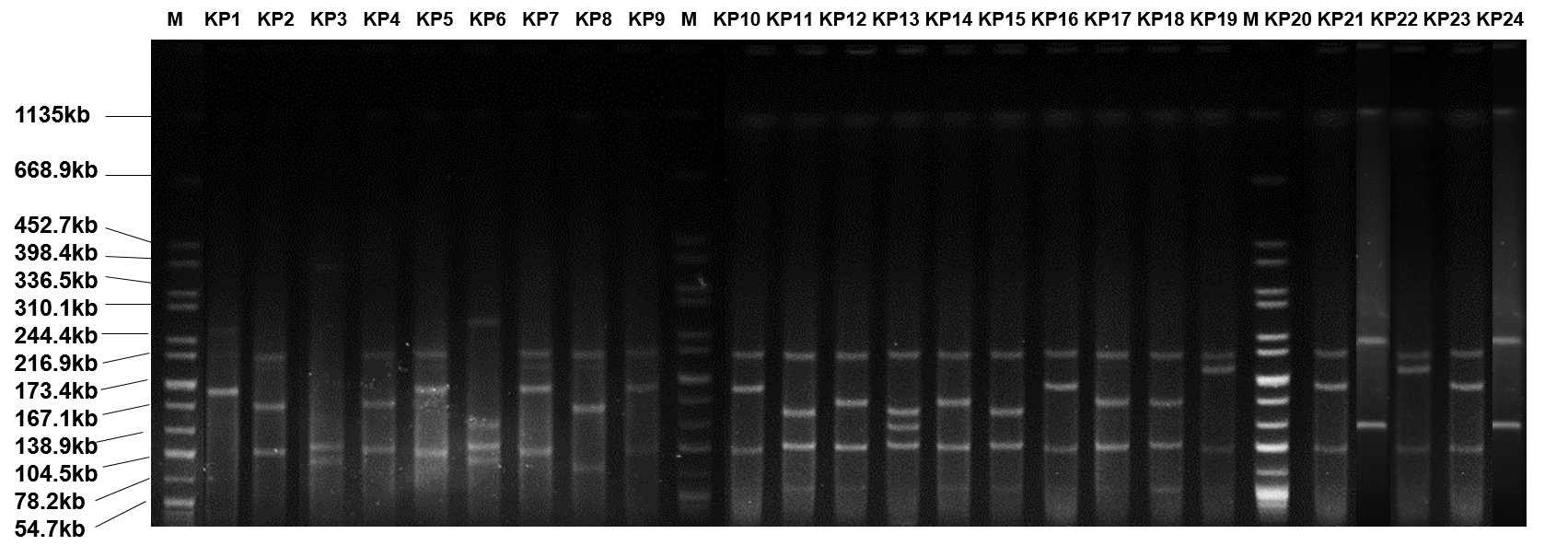


Figure S1. The S1-PFGE of 24 carbapenem-resistant Klebsiella pneumoniae strains

Notes: S1 nuclease digestion of genomic DNA of *K. pneumoniae* strains was followed by PFGE. Plasmid bands are shown as linearized fragment on the gel.Lane M, reference standard strain Salmonella serotype Braenderup H9812 restricted with Xbal.
